# New Endothelial Mechanisms in Glomerular (Patho)biology and Proteinuria Development Captured by Intravital Multiphoton Imaging

**DOI:** 10.3389/fmed.2021.765356

**Published:** 2021-10-13

**Authors:** Georgina Gyarmati, Chaim O. Jacob, János Peti-Peterdi

**Affiliations:** ^1^Departments of Physiology and Neuroscience, and Medicine, Zilkha Neurogenetic Institute, University of Southern California, Los Angeles, CA, United States; ^2^Division of Rheumatology and Immunology, Department of Medicine, University of Southern California, Los Angeles, CA, United States

**Keywords:** proteinuria, podocyte, glomerular endothelium, glycocalyx, immune cells, lupus nephritis

## Abstract

In the past two decades, intravital imaging using multiphoton microscopy has provided numerous new visual and mechanistic insights into glomerular biology and disease processes including the function of glomerular endothelial cells (GEnC), podocytes, and the development of proteinuria. Although glomerular endothelial injury is known to precede podocyte damage in several renal diseases, the primary role of GEnCs in proteinuria development received much less attention compared to the vast field of podocyte pathobiology. Consequently, our knowledge of GEnC mechanisms in glomerular diseases is still emerging. This review highlights new visual clues on molecular and cellular mechanisms of GEnCs and their crosstalk with podocytes and immune cells that were acquired recently by the application of multiphoton imaging of the intact glomerular microenvironment in various proteinuric disease models. New mechanisms of glomerular tissue remodeling and regeneration are discussed based on results of tracking the fate and function of individual GEnCs using serial intravital multiphoton imaging over several days and weeks. The three main topics of this review include (i) the role of endothelial injury and microthrombi in podocyte detachment and albumin leakage via hemodynamic and mechanical forces, (ii) the alterations of the endothelial surface layer (glycocalyx) and its interactions with circulating immune cells in lupus nephritis, and (iii) the structural and functional remodeling and regeneration of GEnCs in hypertension, diabetes, and other experimental injury conditions. By the comprehensive visual portrayal of GEnCs and the many other contributing glomerular cell types, this review emphasizes the complexity of pathogenic mechanisms that result in proteinuria development.

## Introduction

Proteinuria is a key clinical marker of kidney dysfunction, and it is commonly due to the disruption of the glomerular filtration barrier (GFB). In the healthy kidney, several traditional and newly recognized layers of the GFB help to prevent the filtration of plasma macromolecules. These include the endothelial surface layer (glycocalyx) and fenestrations, the glomerular basement membrane (GBM) and the podocyte foot processes including their slit diaphragm (1, 2).

Research in the past 20 years centered mainly on the podocyte and led to major advances in understanding the numerous pathogenic molecular mechanisms in the slit diaphragm largely thanks to advances in mouse and human genetics (3). However, intercellular communications in the glomerulus including the role of glomerular endothelial cells (GEnCs) in proteinuria development received much less attention. Although GEnC injury is a known early event preceding podocyte pathomechanisms in several glomerular pathologies (4, 5), the primary roles and contributions of GEnCs and their crosstalk to podocytes in proteinuria development are less understood.

**Figure 1:**
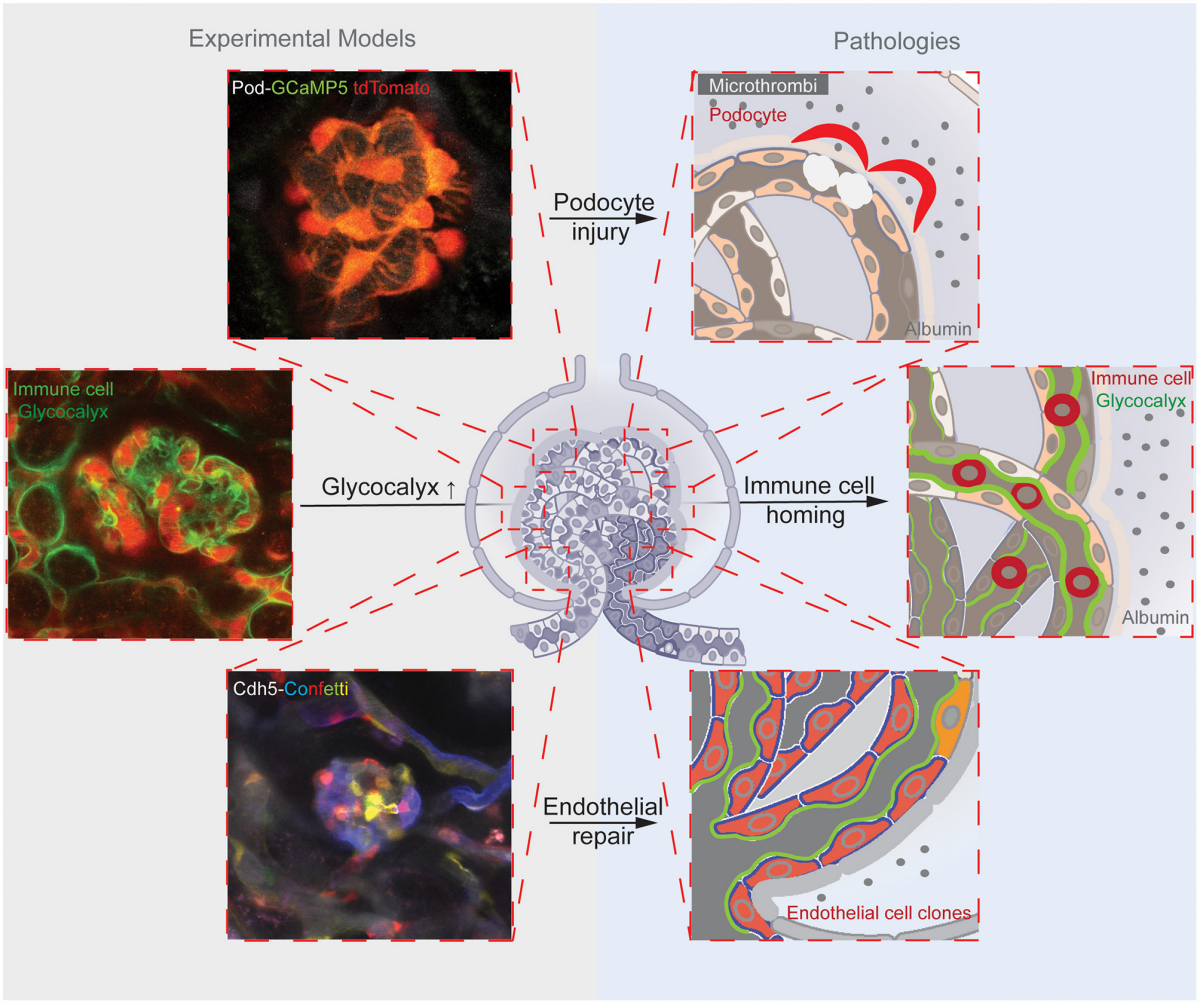
Illustrations of the reviewed three main glomerular endothelial mechanisms (shown in the top, center, and bottom rows). Corresponding representative images of the experimental models (on the left) that were applied to study the pathological mechanisms (schematics on the right). The top panels illustrate the endothelial injury-induced formation of microthrombi and hemodynamic and mechanical GFB alterations leading to podocyte damage, and mice with podocyte-specific expression of the calcium reporter GCaMP5 (green)/tdTomato (red). The center panels show the endothelial surface layer (glycocalyx)-mediated glomerular homing of immune cells, and the labeling of the glomerular endothelial glycocalyx with FITC-WGA and immune cells with anti-CD44-Alexa Fluor 488 antibodies (green) and the circulating albumin with Alexa Fluor 594 (red). The bottom panels demonstrate clonal remodeling and functional regeneration of the glomerular endothelium by local endothelial precursor cells, and the genetic Cdh5-Confetti mouse model with multicolor fluorescent reporter (CFP/GFP/YFP/RFP, a.k.a. Confetti) expression in GEnCs.

**Figure 2:**
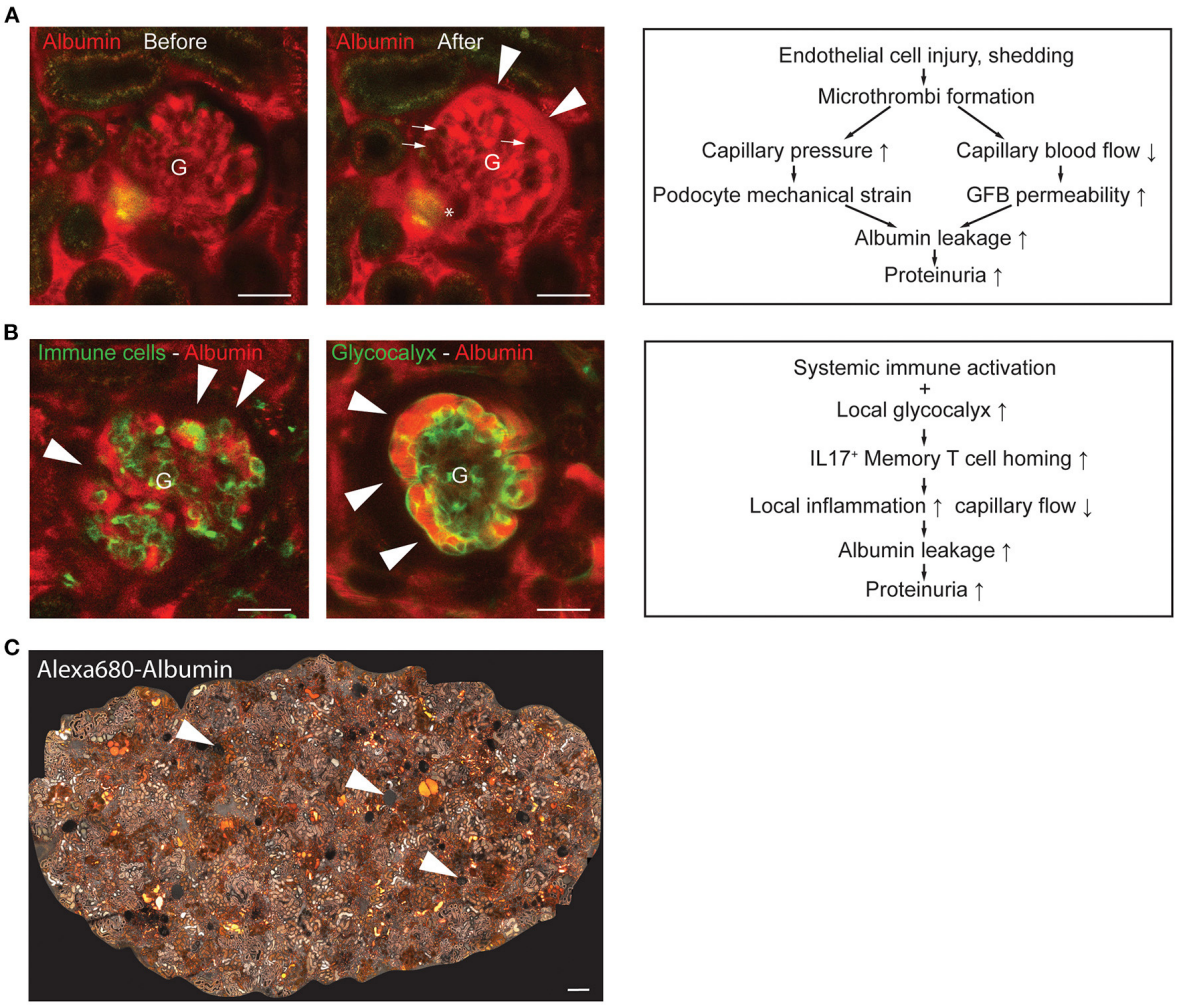
Representative intravital MPM images of glomerular endothelial injury models and schematics of their underlying pathogenic mechanisms. **(A)** Laser-induced injury of GEnCs [injury site indicated by an asterisk (*)] triggered microthrombi formation in several glomerular capillary segments [dark, excluding the plasma marker Albumin-Alexa Fluor 594 (red), indicated by arrows] and robust albumin leakage into the Bowman's space (arrowheads). Note the normal flow of red blood cells (dark, plasma excluding) in glomerular (G) capillaries before GEnC laser injury (left panel), and the blockade of red blood cell passage due to microthrombi with only reduced plasma flow (intense red) in glomerular capillaries after laser injury (middle panel). The “after” image of the same glomerulus was acquired 1 min following the “before” image; the entire video sequence starting at the end of laser injury is shown in Supplementary Video 1 available at https://figshare.com/s/0ca6f8d0dae48415ca01. Schematic illustration of the mechanisms involved in the development of GEnC injury-induced albumin leakage and proteinuria development (right panel). **(B)** Representative *in vivo* MPM images of the NZM.2328 BAFF transgenic mouse model of lupus nephritis (LN). Endogenous circulating CD44^+^ immune cells were labeled with iv injected anti-CD44-Alexa Fluor 488 antibodies (green) and the circulating plasma albumin with Alexa Fluor 594 (red) (left panel). Note the leakage of plasma albumin into the Bowman's space (arrowheads) based on the light red color of the Bowman's space in contrast to the dark (albumin-free) Bowman's space shown in panel A above. Alternatively, the GEnC glycocalyx was labeled by iv injected FITC-WGA (green, arrowheads) (middle panel). Schematic illustration of the mechanisms involved in glomerular immune cell homing and proteinuria development in LN (right panel). **(C)** Representative tile scan image of the entire superficial cortical area of an 8-months old NZM.2328 mouse kidney injected with Albumin-Alexa Fluor 680 iv (shown in grayscale) illustrating the complexity of LN pathologies. These include glomerular albumin leakage (arrowheads, based on the light gray color of the Bowman's space), high albumin-containing tubule segments (intense white), and several focal fibrotic interstitial areas (lack of glomerular or tubular structures). Bars are 50 μm in **(A,B)** and 100 μm in **(C)**.

Intravital imaging using multiphoton microscopy (MPM) has become a revolutionary new research approach in the field that in the past 20 years contributed significantly to our understanding of glomerular and tubular mechanisms of proteinuria (6–9). It should be noted that the earliest kidney MPM studies were technically limited to imaging the most superficial glomeruli in mostly non-physiological models using specific strains (e.g., Munich-Wistar-Fromter rat) or experimental manipulations to generate superficial glomeruli in mice (9–13). A few studies discussed in this review used these earlier approaches (14–16), therefore those results on GFB function should be interpreted with caution. However, recent major improvements in laser and microscopy technologies have made it possible to routinely image deep cortical glomeruli in the intact mouse kidney (17). Most of the new knowledge and topics reviewed here were derived from using these recent state-of-the-art MPM imaging approaches in intact adult mouse kidneys up to 8-months of age (Figures 1, 2) (18–22). Importantly, MPM has been able to directly visualize (patho)physiological processes of the entire glomerulus, the many elements of the GFB simultaneously as parts of the whole functional unit rather than just focusing on a single cell type (12, 14). This integrative and holistic pathophysiology approach has successfully identified multiple levels of cell-to-cell interactions between individual cells and cell types of the GFB. Intravital imaging was able to shed light on the consequences of manipulating a single GFB layer on overall glomerular structure and function including albumin leakage (10, 12, 14, 15, 18, 20, 21). This review highlights important functions and roles of GEnCs in the development of glomerular injury and proteinuria based on recent *in vivo* MPM studies in both physiological and disease models (Figure 1). In addition, the targeting of newly discovered pathophysiological mechanisms for potential new regenerative therapeutic developments for proteinuric kidney diseases is also explored.

## Endothelial Injury, Microthrombi, Hemodynamic, and Mechanical Factors in Albumin Leakage

GEnCs play key roles in the physiological function and maintenance of the healthy GFB and in the development of glomerular pathologies. The unique morphological and functional features of GEnCs include their flat and fenestrated profile that enables the normally enormous rate of glomerular plasma ultrafiltration (23), and the presence of a dense and negatively charged endothelial surface layer (glycocalyx) that constitutes a newly recognized layer of the GFB (2, 24). Rich in proteoglycans and secreted glycosaminoglycans (e.g., heparan sulfate and hyaluronic acid), the GEnC glycocalyx has important roles in several processes including restricting albumin passage through the GFB, binding chemokines and growth factors, and immune cell adhesion (2, 24, 25). Intravital MPM imaging approaches have successfully visualized these GEnC structures and functions in the intact living kidney including the bulk fluid flow in fenestrated capillaries (11, 26) and the presence and alterations in GEnC glycocalyx in various disease models (15, 19–21).

Regardless of the type of GEnC injury, capillary occlusion and reduced plasma flow (based on internal vascular obstruction) were the common pathological and hemodynamic features of the glomerular capillaries observed by MPM imaging. In the rat puromycin (PAN)-induced focal segmental glomerulosclerosis (FSGS) model this was due to shedding of individual GEnCs (likely the result of direct GEnC cytotoxicity) and the instantaneous formation of localized microthrombi in the affected capillary area (14). Similarly, laser-induced mild injury of single GEnCs immediately led to localized microthrombi formation that reduced capillary segment perfusion, blocked red blood cell passage but allowed diminished plasma flow (12). Temporary or permanent adhesion of circulating immune cell types triggered similar hemodynamic alterations (21, 27). Importantly, capillary segment obstruction and altered local plasma flow in all injury models triggered the development of albumin leakage through the GFB and proteinuria (12, 14, 21). As described in these earlier reports and exemplified in Figure 2A (also in Supplementary Video 1 at https://figshare.com/s/0ca6f8d0dae48415ca01), robust albumin leakage into the Bowman's space can develop within 1 min of thrombotic occlusion of a few glomerular capillary segments. On one hand, the rapidly increasing GFB albumin permeability was causatively linked to mechanical factors in the capillary segments that localized pre-occlusion, such as increased capillary pressure, fluid filtration and shear stress of podocyte foot processes, and increased podocyte mechanical strain that ultimately led to podocyte detachment (Figure 2A) (14). On the other hand, the increased GFB albumin permeability in capillary segments that localized after the occlusion can be explained by reduced GBM compression [according to the gel compression model (28)] and/or the reduced capillary blood flow causing locally reduced filtration, which in turn can diminish the electrical field (streaming potential) that normally helps to restrict albumin passage according to the electrokinetic model (29, 30). Regardless of the multiple mechanistic alternatives listed above, the primary event was always GEnC injury underscoring the major importance of the glomerular endothelium in proteinuria development.

## Endothelial Surface Layer (glycocalyx) Controls Glomerular Homing of Immune Cells and Proteinuria

The glomerular endothelial surface layer (glycocalyx) is a newly recognized layer of the GFB and functions as a major determinant of GFB macromolecule permeability (2, 24). In addition, it is essential in immune cell adhesion (25). According to the existing paradigm, the negatively charged glycocalyx covering the GEnC fenestrations acts as a barrier against albumin filtration, and therefore treatment with glycocalyx degrading enzymes induces albumin passage across the endothelium (15, 31). On the other hand, components of the glycocalyx (e.g., heparan sulfate and hyaluronic acid) play well-known anchoring roles in immune cell homing, and therefore trigger local inflammation that increases albumin permeability (25). The key to understanding the ultimate role of the glomerular glycocalyx may be in the balance between these pro and anti-proteinuric functions.

Our recent intravital MPM imaging study visually captured the essential role of the GEnC glycocalyx in the pathogenesis of lupus nephritis (21). Unexpectedly, MPM imaging found a robust accumulation rather than loss of the GEnC glycocalyx, and a high level of glomerulus-specific homing of CD44^+^-IL17^+^ activated memory T cells in two different proteinuric LN mouse models (Figures 2B,C) (21). This glycocalyx accumulation involved its hyaluronic acid component and appeared to be specific to LN, since other inflammatory conditions such as diabetes were associated with diminished glycocalyx as described before (16, 21). Glomerular immune cell homing, local inflammation, glomerular albumin leakage, and albuminuria observed in these LN mouse models were mediated via the binding of CD44 (expressed on the surface of activated memory T cells) to its ligand hyaluronic acid present in excess in the GEnC glycocalyx (21). Importantly, treatment with different glycocalyx degrading enzymes reduced glycocalyx thickness back to normal levels (rather than completely eliminating it), disrupted immune cell homing, improved clinical LN including albuminuria and animal survival (21). One important conclusion from these studies was that GEnC glycocalyx thickness is not in linear relationship with proteinuria development, and too much of a generally protective mechanism (glycocalyx) can be pathogenic. In other words, the role of GEnC glycocalyx in proteinuria development is complex and involves both protective and pathogenic mechanisms. The other important outcome of this study was the efficient therapeutic targeting of the excess GEnC glycocalyx by hyaluronidase that may be further developed for LN.

## Endothelial Repair and Vascular Regeneration Improve Albumin Leakage

Improving our understanding of the dynamics and mechanisms of GEnC turnover and repair after injury at the single-cell level in the intact living kidney over time is critically important for the development of mechanism-based regenerative therapeutic approaches for glomerular kidney diseases. The research technique of serial intravital MPM imaging of the same glomeruli in the same mouse kidney over several days and weeks has been developed and used earlier in combination with cell function and genetic cell fate tracking tools to examine glomerular tissue remodeling by podocytes (10) and cells of the renin lineage (22). This same approach was recently applied to GEnCs with single-cell resolution to shed light on the dynamic and functional endothelial remodeling mechanisms and vascular regeneration in healthy glomeruli and in disease models. Compared to the slow turnover observed in other organs and vascular beds, the rapid, and clonal expansion of single GEnC precursors were quantitatively visualized in response to hypertensive, diabetic, and laser-induced GEnC injuries (20). Interestingly, GEnC progenitor cells were locally residing at the glomerular vascular pole, mostly in the terminal afferent and/or efferent arteriole segments rather than derived from a circulating progenitor pool. Functionally, the newly and clonally remodeled (regenerated) glomerular capillary segments featured a lower amount of GEnC glycocalyx and a lower level of GFB albumin permeability compared to non-clonal segments. These morphological and functional features were consistent with functionally regenerated capillaries and/or with a less differentiated state of GEnCs. Activating and enhancing the function of this newly identified GEnC progenitor cell population may facilitate glomerular vascular regeneration in future therapeutic development.

## Discussion

The recent intravital MPM imaging studies reviewed here shed new light on the many important roles and contributions of GEnCs to glomerular pathobiology and proteinuria development. The numerous technical advances applied in these investigations were instrumental to successfully label and directly and quantitatively visualize using MPM imaging the glomerular endothelium at the single-cell level, their subcellular features and functions including glycocalyx output, immune cell interactions, fenestrations, ultrafiltration, and albumin permeability in the intact living kidney. The simultaneous imaging of the structure and function of all GFB layers provided visual evidence for the important and primary roles of the glomerular endothelium in several mechanisms of proteinuria development in addition to the well-known roles played by the GBM and podocytes. In addition, the many contributing factors that MPM imaging studies revealed, including microthrombi, locally altered hemodynamics and mechanical strain, glycocalyx, immune cell homing, and endothelial remodeling altogether emphasizes the complexity of pathogenic mechanisms that result in proteinuria. Finally, the newly identified GEnC molecular and cellular mechanisms are promising therapeutic targets for glomerular diseases.

## Author Contributions

GG, CJ, and JP-P wrote and prepared the manuscript for publication. All authors contributed to the article and approved the submitted version.

## Funding

This work was supported by US National Institutes of Health grants DK123564 and S10OD021833 to JP-P and AR057172 to CJ.

## Conflict of Interest

The authors declare that the research was conducted in the absence of any commercial or financial relationships that could be construed as a potential conflict of interest.

## Publisher's Note

All claims expressed in this article are solely those of the authors and do not necessarily represent those of their affiliated organizations, or those of the publisher, the editors and the reviewers. Any product that may be evaluated in this article, or claim that may be made by its manufacturer, is not guaranteed or endorsed by the publisher.

## References

[1] Deen WM Lazzara MJ Myers BD. Structural determinants of glomerular permeability. Am J Physiol Renal Physiol. (2001) 281:F579–96. 10.1152/ajprenal.2001.281.4.F579 11553505

[2] Salmon AH Neal CR Harper SJ. New aspects of glomerular filtration barrier structure and function: five layers (at least) not three. Curr Opin Nephrol Hypertens. (2009) 18:197–205. 10.1097/MNH.0b013e328329f837 19365184

[3] Feng D. Phosphorylation of key podocyte proteins and the association with proteinuric kidney disease. Am J Physiol Renal Physiol. (2020) 319:F284–91. 10.1152/ajprenal.00002.2020 32686524 PMC7528399

[4] Sedrakyan S Villani V Da Sacco S Tripuraneni N Porta S Achena A. Amniotic fluid stem cell-derived vesicles protect from VEGF-induced endothelial damage. Sci Rep. (2017) 7:16875. 10.1038/s41598-017-17061-2 29203902 PMC5715019

[5] Sun YB Qu X Zhang X Caruana G Bertram JF Li J. Glomerular endothelial cell injury and damage precedes that of podocytes in adriamycin-induced nephropathy. PLoS ONE. (2013) 8:e55027. 10.1371/journal.pone.0055027 23359116 PMC3554670

[6] Gyarmati G Kadoya H Moon JY Burford JL Ahmadi N Gill IS. Advances in renal cell imaging. Semin Nephrol. (2018) 38:52–62. 10.1016/j.semnephrol.2017.09.004 29291762 PMC5773263

[7] Martins JR Haenni D Bugarski M Polesel M Schuh C Hall AM. Intravital kidney microscopy: entering a new era. Kidney Int. (2021) 100:527–35. 10.1016/j.kint.2021.02.042 34015315

[8] Peti-Peterdi J Kidokoro K Riquier-Brison A. Novel *in vivo* techniques to visualize kidney anatomy and function. Kidney Int. (2015) 88:44–51. 10.1038/ki.2015.65 25738253 PMC4490063

[9] Sandoval RM Molitoris BA. Intravital multiphoton microscopy as a tool for studying renal physiology and pathophysiology. Methods. (2017) 128:20–32. 10.1016/j.ymeth.2017.07.014 28733090 PMC5730351

[10] Hackl MJ Burford JL Villanueva K Lam L Suszták K Schermer B. Tracking the fate of glomerular epithelial cells *in vivo* using serial multiphoton imaging in new mouse models with fluorescent lineage tags. Nat Med. (2013) 19:1661–6. 10.1038/nm.3405 24270544 PMC3884556

[11] Kang JJ Toma I Sipos A McCulloch F Peti-Peterdi J. Quantitative imaging of basic functions in renal (patho)physiology. Am J Physiol Renal Physiol. (2006) 291:F495–502. 10.1152/ajprenal.00521.2005 16609147

[12] Peti-Peterdi J Sipos A. A high-powered view of the filtration barrier. J Am Soc Nephrol. (2010) 21:1835–41. 10.1681/ASN.2010040378 20576805 PMC4581726

[13] Sandoval RM Wagner MC Patel M Campos-Bilderback SB Rhodes GJ Wang E. Multiple factors influence glomerular albumin permeability in rats. J Am Soc Nephrol. (2012) 23:447–57. 10.1681/ASN.2011070666 22223875 PMC3294301

[14] Burford JL Gyarmati G Shirato I Kriz W Lemley KV Peti-Peterdi J. Combined use of electron microscopy and intravital imaging captures morphological and functional features of podocyte detachment. Pflugers Arch. (2017) 469:965–74. 10.1007/s00424-017-2020-0 28664407 PMC5553195

[15] Salmon AH Ferguson JK Burford JL Gevorgyan H Nakano D Harper SJ. Loss of the endothelial glycocalyx links albuminuria and vascular dysfunction. J Am Soc Nephrol. (2012) 23:1339–50. 10.1681/ASN.2012010017 22797190 PMC3402289

[16] Satoh M Kobayashi S Kuwabara A Tomita N Sasaki T Kashihara N. *In vivo* visualization of glomerular microcirculation and hyperfiltration in streptozotocin-induced diabetic rats. Microcirculation. (2010) 17:103–12. 10.1111/j.1549-8719.2009.00010.x 20163537

[17] Schuh CD Haenni D Craigie E Ziegler U Weber B Devuyst O. Long wavelength multiphoton excitation is advantageous for intravital kidney imaging. Kidney Int. (2016) 89:712–9. 10.1038/ki.2015.323 26509590

[18] Burford JL Villanueva K Lam L Riquier-Brison A Hackl MJ Pippin J. Intravital imaging of podocyte calcium in glomerular injury and disease. J Clin Invest. (2014) 124:2050–8. 10.1172/JCI71702 24713653 PMC4001540

[19] Butler MJ Ramnath R Kadoya H Desposito D Riquier-Brison A Ferguson JK. Aldosterone induces albuminuria via matrix metalloproteinase-dependent damage of the endothelial glycocalyx. Kidney Int. (2019) 95:94–107. 10.1016/j.kint.2018.08.024 30389198 PMC6506575

[20] Desposito D Schiessl IM Gyarmati G Riquier-Brison A Izuhara AK Kadoya H. Serial intravital imaging captures dynamic and functional endothelial remodeling with single-cell resolution. JCI Insight. (2021) 6:e123392. 10.1172/jci.insight.123392 33848265 PMC8262275

[21] Kadoya H Yu N Schiessl IM Riquier-Brison A Gyarmati G Desposito D. Essential role and therapeutic targeting of the glomerular endothelial glycocalyx in lupus nephritis. JCI Insight. (2020) 5:e131252. 10.1172/jci.insight.131252 32870819 PMC7566710

[22] Kaverina NV Kadoya H Eng DG Rusiniak ME Sequeira-Lopez ML Gomez RA. Tracking the stochastic fate of cells of the renin lineage after podocyte depletion using multicolor reporters and intravital imaging. PLoS ONE. (2017) 12:e0173891. 10.1371/journal.pone.0173891 28329012 PMC5362207

[23] Ballermann BJ. Contribution of the endothelium to the glomerular permselectivity barrier in health and disease. Nephron Physiol. (2007) 106:p19–25. 10.1159/000101796 17570944

[24] Satchell S. The role of the glomerular endothelium in albumin handling. Nat Rev Nephrol. (2013) 9:717–25. 10.1038/nrneph.2013.197 24080802

[25] Dane MJ van den Berg BM Lee DH Boels MG Tiemeier GL Avramut MC. A microscopic view on the renal endothelial glycocalyx. Am J Physiol Renal Physiol. (2015) 308:F956–66. 10.1152/ajprenal.00532.2014 25673809

[26] Rosivall L Mirzahosseini S Toma I Sipos A Peti-Peterdi J. Fluid flow in the juxtaglomerular interstitium visualized *in vivo*. Am J Physiol Renal Physiol. (2006) 291:F1241–7. 10.1152/ajprenal.00203.2006 16868308

[27] Devi S Li A Westhorpe CL Lo CY Abeynaike LD Snelgrove SL. Multiphoton imaging reveals a new leukocyte recruitment paradigm in the glomerulus. Nat Med. (2013) 19:107–12. 10.1038/nm.3024 23242472

[28] Fissell WH Miner JH. What is the glomerular ultrafiltration barrier? J Am Soc Nephrol. (2018) 29:2262–4. 10.1681/ASN.2018050490 30030419 PMC6115656

[29] Hausmann R Kuppe C Egger H Schweda F Knecht V Elger M. Electrical forces determine glomerular permeability. J Am Soc Nephrol. (2010) 21:2053–8. 10.1681/ASN.2010030303 20947631 PMC3014018

[30] Moeller MJ Tenten V. Renal albumin filtration: alternative models to the standard physical barriers. Nat Rev Nephrol. (2013) 9:266–77. 10.1038/nrneph.2013.58 23528417

[31] Dane MJ van den Berg BM Avramut MC Faas FG van der Vlag J Rops AL. Glomerular endothelial surface layer acts as a barrier against albumin filtration. Am J Pathol. (2013) 182:1532–40. 10.1016/j.ajpath.2013.01.049 23518410

